# Support Needs of Parents of Children With Congenital Anomalies Across Europe: A EUROlinkCAT Survey

**DOI:** 10.1111/cch.70160

**Published:** 2025-09-05

**Authors:** Elena Marcus, Anna Latos‐Bielenska, Anna Jamry‐Dziurla, Ingeborg Barišić, Clara Cavero‐Carbonell, Elly Den Hond, Ester Garne, Lucas Genard, Ana João Santos, L. Renée Lutke, Carlos Matias Dias, Lucía Páramo‐Rodríguez, Christina Neergaard Pedersen, Amanda J. Neville, Annika Niemann, Ljubica Odak, Anna Pierini, Anke Rissmann, Judith Rankin, Joan K. Morris

**Affiliations:** ^1^ Population Health Research Institute St George's, University of London London UK; ^2^ Chair and Department of Medical Genetics Poznan University of Medical Sciences, Collegium Maius Poznań Poland; ^3^ Children's Hospital Zagreb, Centre of Excellence for Reproductive and Regenerative Medicine Medical School University of Zagreb Zagreb Croatia; ^4^ Rare Diseases Research Unit Foundation for the Promotion of Health and Biomedical Research in the Valencian Region Valencia Spain; ^5^ Provincial Institute for Hygiene (PIH) Antwerpen Belgium; ^6^ Family Medicine and Population Health (FAMPOP) University of Antwerp, Gouverneur Kingsbergencentrum Wilrijk Belgium; ^7^ Department of Paediatrics and Adolescent Medicine, Lillebaelt Hospital University Hospital of Southern Denmark Kolding Denmark; ^8^ Epidemiology Department National Institute of Health Doctor Ricardo Jorge Lisbon Portugal; ^9^ Department of Genetics University Medical Center, University of Groningen Groningen the Netherlands; ^10^ IMER Registry (Emilia Romagna Registry of Birth Defects) University of Ferrara and Azienda Ospedaliero Universitaria di Ferrara Ferrara Italy; ^11^ Malformation Monitoring Centre Saxony‐Anhalt, Medical Faculty Otto‐von‐Guericke‐University Magdeburg Magdeburg Germany; ^12^ Unit of Epidemiology of Rare Diseases and Congenital Anomalies Institute of Clinical Physiology, National Research Council Pisa Italy; ^13^ Population Health Sciences Institute Newcastle University Newcastle upon Tyne UK

**Keywords:** congenital anomaly, paediatric, parents, support needs, survey

## Abstract

**Background:**

Parents and carers of children with congenital anomalies can experience stress when managing their child's healthcare needs. It is important that they are well supported. This study explored the support needs of parents/carers of children with a congenital anomaly across Europe.

**Methods:**

We developed a cross‐sectional online survey to measure parents' experiences of support at diagnosis and in subsequent years. We recruited parents/carers of children (0–10 years) with cleft lip, congenital heart defect requiring surgery, Down syndrome and/or spina bifida, online via relevant organisations in 10 European countries (March–July 2021).

**Results:**

A total of 1109 parents/carers were recruited in Poland (*n* = 476), the United Kingdom (*n* = 120), Germany (*n* = 97), Belgium/Netherlands (*n* = 74), Croatia (*n* = 68), Italy (*n* = 59), other European countries (*n* = 92) and unspecified/non‐European countries (*n* = 84). At diagnosis, only 27% (262/984) of parents/carers reported feeling well supported by HCPs, and 49% (468/959) reported that they would have liked professional psychological support but did not receive it. After diagnosis, satisfaction with support from HCPs differed significantly across countries, whereas satisfaction with support from participants' personal networks was more consistent.

**Conclusion:**

Our findings suggest that parents require greater support from HCPs at diagnosis, particularly psychological support. Further research in a European context is needed to understand what the barriers to support might be and how it may be integrated more effectively into existing healthcare systems.

## Summary

- This study found a high level of unmet psychological support for parents of children with congenital anomalies at diagnosis.
- Implementing routine screening for psychological distress at the time of a child's diagnosis of a congenital anomaly could help identify parents in need of additional psychosocial support.
- Further research exploring the barriers and facilitators to psychological support for families in Europe would be beneficial.

## 1 Introduction

Congenital anomalies (CAs) are a leading cause of disability in infants and children in high‐income countries (Euro‐Peristat Project 2018). In Europe, major CAs were estimated to affect 24 per 1000 births between 2003 and 2007, of which 80% resulted in a live birth (Dolk et al. 2010). When parents receive a diagnosis of a CA, they can experience a period of shock, sadness and fear, as they try to make sense of unexpected news (Carlsson et al. 2017; Nelson et al. 2012; Kasparian et al. 2016). Many children with CAs require frequent healthcare visits (Bishop et al. 2018; Urhoj et al. 2022), and some may also need additional medical care provided in the home (Oakley et al. 2021). Balancing these ongoing needs with usual family life and employment (Lemacks et al. 2013) can affect parents physically, emotionally and financially (Stock et al. 2024; Biber et al. 2019; Rutter et al. 2024) and impact their quality of life (Garcia Rodrigues et al. 2022).

There are different types of support that parents may benefit from. These include reliable and empathic advice from healthcare professionals (HCPs) (Carlsson et al. 2015), practical support from friends and family (e.g., looking after siblings) (Carlsson et al. 2017; Bratt et al. 2015) and emotional support from mental health professionals (Holm et al. 2021; McCorkell et al. 2012). Patient/parent organisations and parents of children with the same condition (peers) also play a particularly important supportive role (Lemacks et al. 2013). It is important that parents have access to such resources, and that these are delivered to a high standard to help reduce parental stress and support family coping and functioning.

In this study, we surveyed parents and carers of children with CAs in 10 European countries about their experiences of support both around the time their child was diagnosed and in subsequent years. We aimed to obtain a general overview of the extent to which parents felt their support needs were being met and to compare findings across countries and CA groups.

## 2 Methods

### 2.1 Study Design and Participants

This was a cross‐sectional online survey that was open to parents, carers or guardians (called *parents* henceforth) of children up to the age of 10, who were diagnosed with cleft lip, spina bifida, congenital heart defect (CHD) which required surgery, and/or Down syndrome. These CAs were chosen to reflect a range of different impairments, including visible (cleft lip) and nonvisible defects (CHD), as well as physical (spina bifida) and learning (Down syndrome) disabilities. Parents were required to live in Europe to participate. The reporting of our findings adheres to the STrengthening the Reporting of OBservational studies in Epidemiology Statement (STROBE) (von Elm et al. 2014) (see Table S2).

### 2.2 Data Collection

#### 2.2.1 Survey

A full description of the survey development is provided elsewhere (Marcus et al. 2022). In summary, the survey was developed in English following a literature review and input from parents, educators, clinicians, and academics with experience in CA research and questionnaire development. The survey was then translated into nine languages, including a forward and back translation under the supervision of researchers within the project to ensure adaptation to local healthcare environments. A full pilot of the final survey was not possible within the project timescales, but the final version was reviewed by five parents and educators in Poland.

The survey was open from 8 March 2021 to 14 July 2021 (see Table 1 for specific dates by country). The survey asked parents to reflect on their experiences of support from different people and organisations at two time points: (1) around the time their child was diagnosed and (2) before the COVID‐19 pandemic (prior to January 2020). We selected this first time point because diagnosis presents a key time for supportive intervention (Carlsson et al. 2017). The second time point was selected because the survey took place in 2021 when the COVID‐19 pandemic was still having an impact on healthcare systems, and we wanted to understand what parents' experiences of support were like prior to this unique period. The survey also included other items about parents' experiences during the pandemic (reported in Latos‐Bielenska et al. 2022), and we wanted participants to be clear about this distinction in time. We did not define the term ‘support’ as we felt it was most important to understand the extent to which parents ‘felt supported’, or ‘felt satisfied with support’, as opposed to trying to quantify the amount or type of support that had been received. This is because the beneficial impacts of support depend on the quality of support, who it is delivered by and other contextual factors (Ekas et al. 2010; Cuzzocrea et al. 2016), which cannot be easily quantified. The survey included the following sections (all survey items are available in the Supporting Information [Section B]):

a Parent demographics (seven items).
b Child demographics and medical information (seven items).
c Support at diagnosis (three items):

- Support from HCPs
- Support from friends/family
- Professional psychological support

d Support after diagnosis (two items):

- Satisfaction with support from eight sources (e.g., specialist doctor or partner)
- Overall need for more support

**TABLE 1:** Recruitment period and participant characteristics by country group.

Characteristic	All	UK	Poland	Germany	Belgium/Netherlands	Croatia	Italy	Other EU^a^
**Recruitment period**								
Start date	—	8 Mar 2021	8 Mar 2021	11 May 2021	19 Apr 2021	26 Apr 2021	16 Jun 2021	6 Apr 2021
End date	—	14 Jul 2021	14 Jul 2021	14 Jul 2021	14 Jul 2021	14 Jul 2021	31 Jul 2021	14 Jul 2021
***N***	986	120	476	97	74	68	59	92
**Relation to child**								
Mother	911 (92%)	116 (97%)	449 (94%)	81 (84%)	64 (86%)	63 (93%)	52 (88%)	86 (95%)
Father	65 (7%)	2 (2%)	24 (5%)	13 (13%)	10 (14%)	5 (7%)	6 (10%)	5 (5%)
Other^b^	8 (1%)	1 (1%)	3 (1%)	3 (3%)	—	—	1 (2%)	—
**Age**								
≤ 30	162 (17%)	18 (15%)	93 (20%)	13 (13%)	15 (20%)	8 (12%)	4 (7%)	11 (12%)
31–40	516 (53%)	53 (45%)	264 (56%)	51 (53%)	35 (47%)	37 (55%)	27 (46%)	49 (53%)
> 40	301 (31%)	47 (40%)	115 (24%)	33 (34%)	24 (32%)	22 (33%)	28 (47%)	34 (35%)
**Education**								
School ≤ 18 years	390 (40%)	44 (37%)	163 (35%)	61 (67%)	44 (60%)	19 (28%)	30 (52%)	29 (32%)
University	482 (49%)	50 (42%)	257 (53%)	27 (29%)	29 (39%)	45 (66%)	19 (33%)	55 (60%)
Post‐graduate	106 (11%)	25 (21%)	56 (11%)	3 (3%)	1 (1%)	4 (6%)	9 (16%)	8 (9%)
**Employment**								
Employed	586 (60%)	81 (68%)	223 (47%)	61 (62%)	61 (82%)	54 (79%)	44 (75%)	62 (69%)
Homemaker/carer	301 (31%)	36 (30%)	198 (42%)	27 (29%)	8 (11%)	7 (10%)	11 (19%)	14 (16%)
Other^c^	94 (9%)	3 (3%)	52 (11%)	9 (9%)	5 (7%)	7 (10%)	4 (7%)	14 (16%)
**Child diagnosis**								
CHD	327 (33%)	49 (40%)	119 (25%)	28 (29%)	28 (38%)	34 (50%)	27 (46%)	42 (46%)
Cleft lip	230 (23%)	12 (10%)	127 (27%)	31 (32%)	30 (40%)	5 (7%)	12 (20%)	13 (14%)
Down syndrome	262 (27%)	46 (38%)	139 (29%)	19 (20%)	5 (7%)	23 (34%)	9 (15%)	21 (23%)
Down syndrome with CHD	55 (6%)	9 (8%)	29 (6%)	8 (8%)	1 (1%)	5 (7%)	1 (2%)	2 (2%)
Spina bifida	112 (11%)	4 (3%)	62 (13%)	11 (11%)	10 (14%)	1 (1%)	10 (17%)	14 (15%)

*Note:* Some subgroup percentages do not add up to 100% due to rounding.

Abbreviation: CHD—congenital heart defect.

^a^ Other European countries: Denmark (*n* = 39), Portugal (*n* = 23), Spain (*n* = 16), Ireland (*n* = 5), Bulgaria (*n* = 2), Albania (*n* = 1), Cyprus (*n* = 1), Lithuania (*n* = 1), Norway (*n* = 1), Romania (*n* = 1), Sweden (*n* = 1), Ukraine (*n* = 1).

^b^ Other family member (*n* = 3), legal guardian related to the child (*n* = 2), legal guardian unrelated to the child (*n* = 3).

^c^ Unemployed (*n* = 56), long‐term sick/disabled (*n* = 17), employed but not working due to Covid‐19 lockdown restrictions (*n* = 12), student (*n* = 8), retired (*n* = 1).

### 2.3 Recruitment

Participants were recruited online using a multi‐modal online convenience sampling strategy. Parents were actively recruited via relevant organisations (see Supporting Information, Section C) in 10 European countries: Belgium, Croatia, Denmark, Germany, Italy, Netherlands, Poland, Portugal, Spain and the United Kingdom. Each organisation posted information about the survey (including a link to the survey) on their website and/or social media (Facebook/Twitter). Table 1 shows the recruitment start and end dates for each participating country.

### 2.4 Statistical Analysis

We conducted descriptive statistics using Stata 17.0 software (StataCorp 2021). Outcomes scored on 4‐point Likert scales were dichotomised (very satisfied/much vs. other responses). We chose to dichotomise the scale in this manner, as we felt that healthcare and other supportive agencies should aim for parents to feel very satisfied with the support they receive. We modelled the data using multivariate logistic regressions including the child's anomaly type and parent's country of residence, age and education level as covariates. The impact of country and anomaly type on outcomes was explored, choosing the largest categories as the comparator groups (Poland and CHD). For age and education, categorical data were collected. For the analysis, age and education were re‐coded into three groups: age (up to 30 years; 31–40 years, over 40 years), education (formal education until 16 or 18 years/technical training; university degree; post‐graduate degree). Age and education were included in our regression models as ordinal variables. To control for multiple comparisons, we adjusted the alpha level to *p* < 0.01 for all analyses. A small proportion of data were missing, and it was unlikely that data were missing at random so we did not adopt more sophisticated multiple imputation techniques. The results section reports adjusted findings only. Unadjusted frequencies for each survey item are presented in the Supporting Information (Section E).

We aimed to recruit 80 participants per country which would have resulted in a power of 80% to determine that a country with 20% of participants replying the highest category (‘very satisfied’ or ‘very much’) (Category 4) was statistically significantly different at the 95% level of significance from a country with 40% of participants replying in Category 4. Due to delays in obtaining ethics approvals, the recruitment target was not met within the timescales for all countries. Data are presented by country if these were available for at least 50 participants. Where there were <50 participants, data were combined into an ‘other European country’ group (which we term henceforth as *Other EU*). This group includes participants from a heterogenous group of countries (see Table 1). Due to similarities in survey responses, geographical location and language, data for Belgium (*n* = 46) and the Netherlands (*n* = 28) were combined into a single group. For CAs, data were categorised according to the four anomalies, and a separate category created for children with Down syndrome and a CHD, as it is common for children with Down syndrome to also have a diagnosis of CHD (Leirgul et al. 2014). There were very small numbers of children with other combinations of the four anomalies (*n* = 15) (e.g., only two children with CHD and spina bifida). We excluded these from the analysis as there were too few to create a meaningful category to explore in our analyses.

We were unable to calculate a response rate. This is because we used a multi‐modal online recruitment strategy and it was not possible to estimate how many potential participants the survey may have reached (McRobert et al. 2018). We report submission rates (i.e., the number of participants who started the survey divided by the number who submitted the survey) (Liu and Wronski 2018), and of those participants who submitted their survey, we report item‐level response rates (the proportion of participants who completed each item) (Bosnjak and Tuten 2001).

### 2.5 Ethics Approval

Ethics approval was granted by the St George’s (University of London) Research Ethics Committee on 18 December 2020 (reference number: 2020.0311). Further local ethics approvals were obtained from each collaborating country, except from the centres in the Netherlands and Denmark whose ethics committees confirmed that no further approvals were needed.

## 3 Results

### 3.1 Participant Characteristics

Overall, 1298 parents started the survey, of whom 1109 (85%) submitted their responses. The submission rate varied across countries, ranging from 78% in Italy to 92% in Belgium and Germany. We excluded 123 (9.5%) submitted surveys from the analysis because: (a) data regarding country of residence were missing (*n* = 80), (b) data about CA type were missing (*n* = 24), (c) participants lived in non‐European countries (*n* = 4), or (d) participants had children with other combinations of the four CAs we included in the study (e.g., cleft lip and spina bifida) (*n* = 15). In total, 986 (89%) participants were included in the analysis.

Most survey respondents were mothers (92%), employed (59%) and aged 31–40 years (71%) (Table 1). Respondents lived in Poland (*n* = 476; 48%), the United Kingdom (*n* = 120; 12%), Germany (*n* = 97; 10%), Belgium/Netherlands (*n* = 74; 8%), Croatia (*n* = 68; 7%) and Italy (*n* = 59; 6%). There were 92 participants in the other EU group (see Table 1 for a list of countries).

The children of the survey respondents were diagnosed with CHD (*n* = 327; 33%), Down syndrome (*n* = 262; 26%), a cleft lip (*n* = 230; 23%), spina bifida (*n* = 112; 11%) and Down syndrome with a CHD (*n* = 55; 6%) (Table 1). A quarter of participants reported that their child had another CA, and 43% reported that their child had at least one other health condition. The largest age group for children was the ‘1–3 years’ group (35% of the sample) and there was a slightly higher proportion of male children (56%).

### 3.2 Support within 1 month of the child's diagnosis

1. Support from HCPs treating your child

**FIGURE 1:**
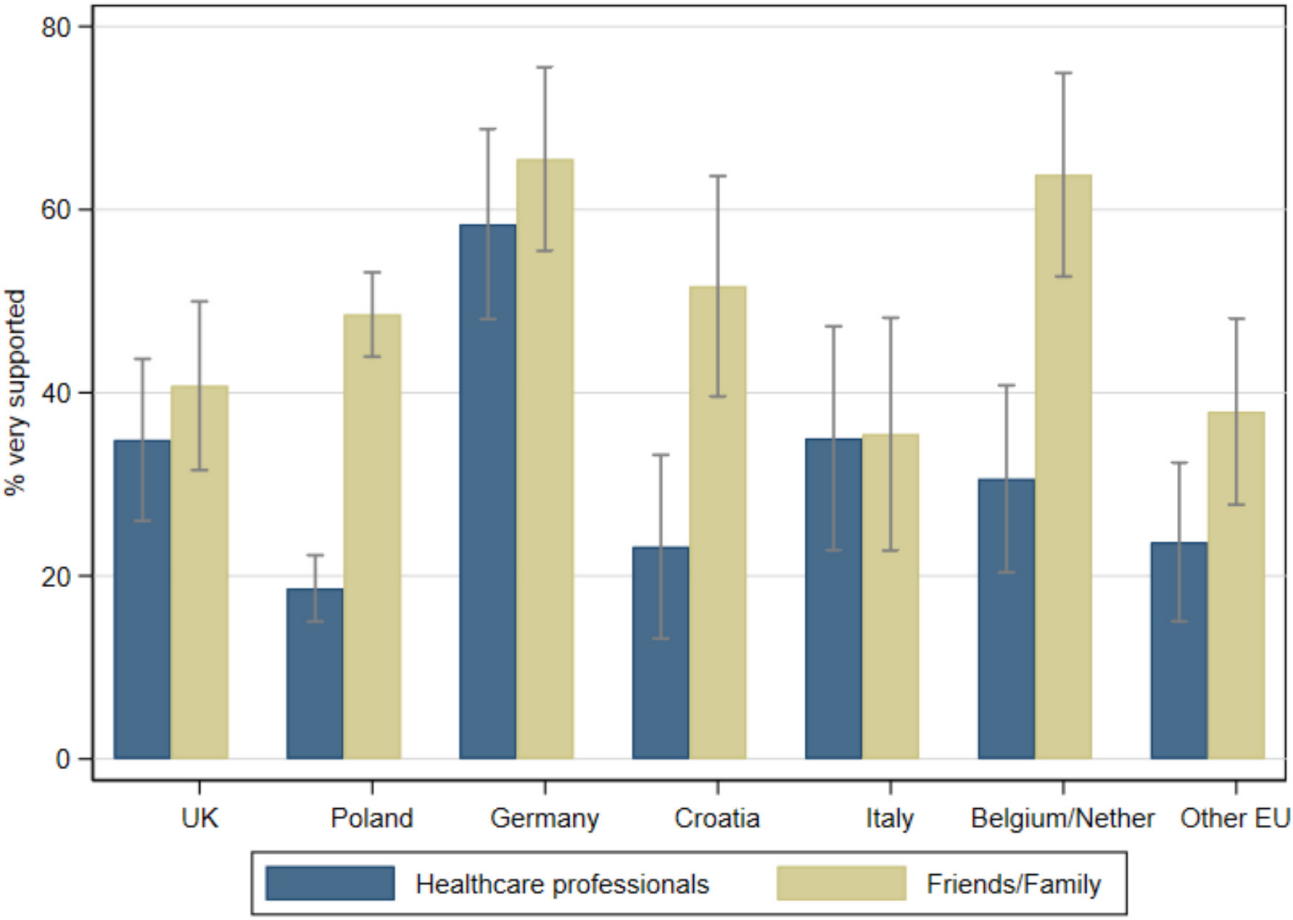
Proportion (adjusted by congenital anomaly type, parental age and education level) of parents reporting that they felt ‘very supported’ by healthcare professionals and friends/family by country, with 95% confidence intervals.

Just over a quarter of participants (27%; 262/984) reported feeling ‘very supported’ by the HCPs treating their child. There was considerable heterogeneity across countries in the extent to which participants felt supported (*p* < 0.001). Poland and Croatia had the lowest proportions of parents reporting feeling ‘very supported’, 19% (95% confidence interval (CI): 15%–22%) and 23% (95% CI: 13%–33%), respectively (see Figure 1). Compared with Poland, significantly more parents in Germany (58%, 95% CI: 48%–69%; *p* < 0.001) and the United Kingdom (35%, 95% CI: 26%–44%; *p* < 0.001) reported feeling ‘very supported’ by HCPs.

2 Support from friends and family

Nearly half of the sample (49%; 469/966) reported feeling supported by friends and family at diagnosis 'very much'. There was significant heterogeneity in this proportion across countries (*p* = 0.002), which was lowest in Italy (35%, 95% CI: 23%–48%) and the other EU group (38%, 95% CI: 28%–48%) (Figure 1). Compared with Poland (49%, 95% CI: 44%–53%), significantly more parents in Germany (66%, 95% CI: 55%–76%; *p* = 0.005) reported feeling ‘very supported’ by their friends and family.

3 Professional psychological support

Overall, nearly half of the sample (49%; 468/959) reported that they would have liked to have received professional psychological support at diagnosis but did not, whereas 11% (111/959) reported that they had received free psychological support, and 4% (36/959) paid for support privately. Just over a third of participants (36%; 344/959) reported that they did not feel they needed any professional psychological support at diagnosis.

Excluding participants who felt they did not need psychological support, overall, 76% (468/615) of participants reported wanting psychological support at diagnosis but not receiving it. There was significant heterogeneity in this figure across countries (*p* = 0.003), with the highest proportions of this unmet need reported in Croatia (87%, 95% CI: 77%–97%) and the United Kingdom (84%, 95% CI: 76%–92%), and the lowest proportion reported in Germany (59%, 95% CI: 45%–73%), which was significantly lower than in Poland (79%, 95% CI: 75%–84%; *p* = 0.004) (see Figure S1).

### 3.3 Support after diagnosis

1. Satisfaction with support from eight sources

Across all countries combined, satisfaction with support ratings were poorest for GPs and schools, with 34% of participants reporting that they were ‘very satisfied’ with support from each of these sources (Table 2). The highest ratings were for participants' partners and peers, with 71% and 66% of participants stating they were ‘very satisfied’ with support from each of these sources, respectively.

- Satisfaction with medical sources (by country)

**TABLE 2:** Proportion of participants reporting that they were ‘very satisfied’ with the support they received from each source, by country.

Country	GP (*N* ^a^ = 679)	Specialist doctor/nurse (*N* ^a^ = 743)	Partner (*N* ^a^ = 752)	Friends/family (*N* ^a^ = 778)	Parents of children with same condition (*N* ^a^ = 624)	Patient organisations (*N* ^a^ = 511)	Schools (*N* ^a^ = 315)
	**%** [95% CI]	**%** [95% CI]	**%** [95% CI]	**%** [95% CI]	**%** [95% CI]	**%** [95% CI]	**%** [95% CI]
Poland	28 [23–33]	34 [27–37]	68 [63–73]	58 [53–63]	71 [66–76]	58 [51–64]	25 [18–33]
UK	26 [17–35]	62 [53–72]	75 [66–84]	51 [41–61]	62 [51–72]	53 [51–63]	49 [35–63]
Germany	59 [46–72]	75 [65–86]	85 [76–94]	73 [62–84]	70 [57–84]	51 [33–69]	38 [22–53]
Croatia	43 [29–57]	58 [45–71]	80 [70–91]	69 [56–81]	72 [60–85]	45 [30–61]	41 [17–65]
Italy	38 [23–52]	45 [31–59]	81 [69–92]	48 [33–62]	45 [30–60]	48 [33–64]	38 [22–53]
Belgium/Netherlands	49 [34–64]	71 [58–83]	64 [52–77]	53 [40–66]	36 [19–53]	9 [0–20]	30 [15–46]
Other EU	49 [35–64]	48 [37–59]	68 [57–79]	49 [38–61]	59 [47–71]	33 [21–44]	37 [22–53]
Total	34 [30–38]	51 [47–54]	71 [68–74]	57 [53–61]	66 [62–69]	50 [45–54]	34 [29–40]
Heterogeneity between countries	*p* = 0.001	*p* < 0.001	*p* = 0.033	*p* = 0.023	*p* = 0.001	*p* = 0.001	*p* = 0.067

*Note:* Adjusted by congenital anomaly type, parental age, and education level. Unadjusted proportions are not included in this table.

Abbreviations: CI = confidence interval; GP = general practitioner.

^a^ Total number of participants completing the item, excluding 'not applicable' responses. Missing data: GP (*n* = 13), specialist doctor/nurse (*n* = 19), partner (*n* = 21), friends/family (*n* = 16), parents of children with the same condition (*n* = 19), patient/parent organisations (*n* = 21), schools (*n* = 47).

There was significant heterogeneity across countries for participants' satisfaction with support from their GPs (*p* = 0.001) and specialist doctors (*p* < 0.001). For GPs, the United Kingdom and Poland had the lowest proportion of ‘very satisfied’ ratings, 26% (95% CI: 17%–35%) and 28% (95% CI: 23%–33%), respectively (Table 2). Compared with Poland, ratings were significantly higher in Germany (59%, 95% CI: 46%–72%; *p* < 0.001), and Belgium/Netherlands (49%, 95% CI: 34%–73%; *p* = 0.005). Poland had the lowest ‘very satisfied’ ratings for specialist doctors/nurses, 34% (95% CI: 27%–37%). Compared with Poland, ratings were significantly greater in the United Kingdom (62%, 95% CI: 53%–72%), Belgium/Netherlands (71%, 95% CI: 58%–83%) and in Germany (75%, 95% CI: 58%–83%).

- Satisfaction with close relationships (by country)

Overall, participants rated their ‘partner’ and ‘friends/family’ relatively highly for support, and there was no significant heterogeneity across countries (64%–85% highly satisfied for ‘partner’; 48%–73% highly satisfied for ‘friends/family’). Participants also rated support from their peers (parents of other children with the same health condition) highly; however, there was significant heterogeneity across countries (*p* = 0.001). Croatia, Poland and Germany had the highest proportions of ‘very satisfied’ ratings for support from peers: 72% (95% CI: 60%–85%), 71% (95% CI: 66%–76%,) and 70% (95% CI: 57%–84%), respectively. Compared with Poland, this percentage was significantly lower in Italy (45%, 95% CI: 30%–60%; *p* = 0.001) and Belgium/Netherlands (36%, 95% CI: 19%–53%; *p* < 0.001).

- Satisfaction with patient/parent organisations and schools (by country)

Overall, 50% (255/515) of participants reported being ‘very satisfied’ with support from patient/parent organisations, although there were considerable differences across countries (*p* = 0.001). Poland had the highest proportion of ‘very satisfied’ ratings for patient/parent organisations (58%, 95% CI: 51%–64%), which were significantly lower in the other EU group (33%, 95% CI:21%–44%) and in Belgium/Netherlands (9%, 95% CI: 0%–20%). Around a third of parents reported being ‘very satisfied’ with support from schools (34%; 109/318) which did not differ significantly across countries.

2 Need for more support

Overall, 36% (298/837) of participants reported that they would have liked more support 'very much'. There was significant heterogeneity in this need across countries (*p* < 0.001). Poland had the highest proportion of participants reporting that they would have liked a lot more support (49%, 95% CI: 44%–54%). This was significantly lower in Croatia (7%, 95% CI: 4%–14%), Belgium/Netherlands (9%, 95% CI: 2%–17%), Italy (18%, 95% CI: 7%–29%), the United Kingdom (24%, 95% CI: 17%–33%) and the other EU group (28%, 95% CI: 18%–37%). Germany was not included in this comparison due to a grammatical error in the item wording which meant the direction of the item was unclear (i.e., if selecting ‘very much’ was a positive or negative answer).

### 3.4 Outcomes by CA type

Overall, there were few differences in outcomes across the five CA groupings. For support at diagnosis, significant heterogeneity across the CA groups was only found for support from HCPs. Around a third (35%; 95% CI: 30%–40%) of parents of children with CHD reported feeling ‘very supported’ by HCPs, compared with only 18% (95% CI:14%–23%) of parents of children with Down syndrome (*p* < 0.001). Satisfaction with support from medical sources, close relationships, patient/parent organisations and schools in subsequent years was also generally similar across the CA groups. Significant heterogeneity between the five CA groups was only found for support from specialist doctors/nurses (*p* < 0.001) and support from peers (*p* = 0.001). For support from specialists, the Down syndrome group had the lowest proportion of ‘very satisfied’ ratings, 36% (95% CI: 29%–42%), which was significantly lower than the CHD group (56%, 95% CI: 50%–63%; *p* < 0.001). For support from peers, the cleft lip group had the highest proportion of parents indicating they were ‘very satisfied’ with support (80%, 95% CI: 73%–86%), which was significantly greater than the CHD group (66%, 95% CI: 59%–72%; *p* = 0.001). Full findings are available in Table S1.

## 4 Discussion

To our knowledge, this is the first quantitative study to compare the lived experiences of parents of children with CAs across Europe. The results of this online European survey highlight four key areas where parents reported insufficient support: general support from HCPs treating their child at the time of diagnosis, psychological support at the time of diagnosis, support from GPs after diagnosis and support from schools.

At the time of diagnosis, only 27% of parents in our sample reported feeling well supported by HCPs and nearly half indicated that they would have liked to have received professional psychological support. These findings are generally consistent with studies from other high‐income countries, which have reported similar levels of unmet need. In an online survey conducted in Australia, Thomas et al. (2023) found that 47% of parents of children with CHD reported a moderate or high need for support in managing stress. In a clinic‐based study in the Netherlands, 50% of mothers and 38% of fathers who received a prenatal diagnosis of an oral cleft expressed a need for professional support (Maarse et al. 2018). In another Dutch clinic‐based survey, Levert et al. (2017) reported that approximately 60% of parents of children with CHD scheduled for cardiac surgery (a particularly stressful period) expressed a general need for psychosocial care. Within this group, 24% of parents of 0‐ to 2‐year‐olds and 13% of parents of 3‐ to 7‐year‐olds reported a specific need for individual psychotherapy. This latter finding is somewhat lower than the 49% observed in our study, possibly due to differences in the timing of data collection; we asked parents to retrospectively reflect on the period surrounding diagnosis, whereas in the study by Levert et al. (2017) parents were administered a questionnaire in clinic when surgery was scheduled. Nonetheless, consistent with our findings, Levert et al. (2017) also found that a third of parents reported no need for additional support.

Beyond diagnosis, satisfaction with GPs was notably low, with only 34% of parents reporting that they were very satisfied with the support received, compared with 51% satisfaction with specialist doctors/nurses. This disparity may reflect GPs' limited knowledge of more specialised health conditions, such as CAs, which can impact their ability to provide relevant and reassuring guidance. It may also be influenced by parents' ability to access GP services. This result was particularly low in the United Kingdom, where challenges in securing GP appointments have been documented (Wise 2024), potentially influencing parents' perception of care quality. In contrast, satisfaction with GPs was highest in Germany, where paediatricians form part of the primary care system and can be consulted directly without a GP referral (Ehrich et al. 2016). In a narrative review, Stock et al. (2024) similarly found that while parents of children with a cleft lip/palate had positive experiences with specialist healthcare teams, they often described nonspecialist HCPs as dismissive, unhelpful and lacking the specific knowledge to support their child's needs.

There were some notable differences in findings across surveyed countries. Satisfaction with support from GPs, specialist doctors/nurses and patient/parent organisations showed significant heterogeneity, whereas satisfaction with support from partners and friends/family was high and consistent across countries. This contrast suggests there may be real differences in the knowledge of HCPs and quality of care delivered to parents across these countries rather than the difference being due to the personal attributes of parents within each group (e.g., having a positive attitude about all sources of support). Parents in Germany, Belgium, and the Netherlands had the highest satisfaction ratings for support from HCPs. Interestingly, parents from Poland and Croatia, who generally reported poorer support from HCPs, had high satisfaction ratings for the support they received from patient/parent organisations and parents of other children with the same health condition as their child (peers). Previous research has shown that parents often turn to informal sources of information when adequate information is not provided by HCPs (Wallace and Mattner 2017; Costa et al. 2019). Our finding may therefore indicate that where support from HCPs is limited, parents may actively seek, or invest time in developing, alternative sources of support such as peer support networks.

Overall, there were few differences in outcomes across the CA groups. The Down syndrome group, however, reported significantly poorer support from HCPs at both time points. In the wider literature, parents of children with Down syndrome have consistently been found to experience less stress and have fewer support needs compared with parents of children with other intellectual disabilities, such as autism or Prader–Willi syndrome (Lanfranchi and Vianello 2012; Lee et al. 2019), a pattern termed the ‘Down syndrome advantage’ (Hodapp et al. 2001). The fact that our study found poorer outcomes in this group compared with the other CA groups (none of which involved other intellectual disabilities) warrants attention, especially if there is a prevailing assumption that this group might have fewer support needs. In fact, in a multicentre European data linkage study, Seaton et al. (2025) found that children born with Down syndrome had higher healthcare needs in the first year of their life compared with children with other CAs, including more frequent hospitalisations and admissions to intensive care. These higher costs are likely due to the increased risk of immune dysfunction in this patient group (Ram and Chinen 2011), with infants and children experiencing more prevalent and severe infections (Santoro et al. 2021; Bloemers et al. 2010). In addition, children with Down syndrome (without a CHD) typically receive fewer planned medical interventions than children with severe CHD or a cleft lip as immediate surgical intervention is often not needed. This may result in fewer quality interactions with specialist teams. The combination of intensive or emergency hospital care needs with less clearly defined care pathways may contribute to lower parental satisfaction in this group.

### 4.1 Implications and Future Research

The diagnosis of a CA is a particularly challenging period for parents (Guiller et al. 2009; Carlsson et al. 2017), and a key target area for psychological and social support interventions (Cuzzocrea et al. 2016). Findings from the present study indicated that there was a high level of unmet psychological support at diagnosis. However, as we assessed this with a single high‐level survey item, it remains unclear whether the lack of support reflects services not being offered, parents not accessing them or other reasons.

There is a gap in research exploring the barriers and facilitators to accessing psychological support for families in Europe. One US‐based study involving parents of children with special healthcare needs found that the most common barriers to psychological support were logistical and financial, with parents reporting that they were unable to undergo treatment due to the cost, a lack of insurance, inconvenient appointment times and locations, as opposed to a lack of a referral (Graaf et al. 2022). Due to differences in the use of medical insurance and availability of psychological support within existing European healthcare systems, it might not be appropriate to extrapolate these findings to the countries explored in our study. Future research would benefit from investigating these barriers and facilitators further in a European setting.

Existing research has examined parental support needs across varying child age groups, conditions, and countries, and there is a lack of studies involving nationally or regionally representative samples. This complicates our ability to accurately estimate the proportion of parents in need of support. Nevertheless, the evidence base consistently points to the existence of a subgroup of parents with significant needs. Identifying these parents is crucial. Considering the high level of unmet need identified in our study, a multifaceted approach is recommended. This includes the routine screening of parents for psychological distress (e.g., for symptoms of stress, anxiety or depression) at key points in their child's healthcare journey, as well as the integration of psychosocial support into standard care pathways. HCPs should receive training to enhance their ability to recognise and respond to signs of emotional distress in family members. Training in empathetic communication would also equip HCPs with the skills to provide supportive care. Finally, HCPs should provide parents with information about parent organisations and locally available peer support networks to promote support beyond clinical settings.

Parents in our study reported relatively low levels of satisfaction with the support provided by schools. Existing evidence indicates that, on average, children with CAs are more likely than their peers to experience academic underachievement, increased school absenteeism and higher rates of special educational needs (Glinianaia et al. 2021; Glinianaia et al. 2024; Fitzsimons et al. 2021). However, the literature on parental support needs within educational settings remains limited (Sedláčková and Kantor 2022), making it challenging to fully contextualise our findings. Given the key role that schools play in the lives of children with CAs and their families, this represents an important gap in the evidence base. Future research should explore this area in greater depth to better understand and inform the development of specific support strategies for families navigating the education system.

## 5 Limitations

A key limitation to the study was the use of a non‐probabilistic convenience sampling design and the reliance on charities and patient/parent organisations to recruit parents and carers via their social media channels. There is therefore a risk of sampling bias. The views of our participants may not be representative of the wider population of parents and carers of children with CAs and may also differ from people who do not tend to engage with these organisations.

Although we recruited a large number of participants overall, the sample sizes for each recruiting country were modest. We used a similar recruitment strategy across countries; however, the length of the recruitment period and number of recruiting organisations differed across countries. This may have affected some of the differences reported; however, we found no clear relationship between recruitment methods and the experiences of parents within each country. It is important to highlight that there may be heterogeneity in the availability of psychological support not only between countries but also within countries, which we have been unable to explore in our analysis. Additionally, we did not ask parents whether their child's diagnosis was communicated during a planned or unplanned consultation. This would likely impact parents' satisfaction with care, as planned settings typically allow for more sensitive and supportive communication, whereas unplanned settings are often more stressful and rushed.

An unexpected finding emerged among parents from Croatia. Along with Poland, they had the lowest proportion of parents reporting they were very supported by HCPs around the time of diagnosis (23%) yet also had the lowest proportion of parents reporting that they needed a lot more support in the period following diagnosis (7%). In contrast, responses from parents in other countries tended to show greater consistency across these two time points. It is possible that this pattern reflects a genuine shift among Croatian parents, from limited support at diagnosis to improved support in the period afterwards. However, it remains unclear why this discrepancy was observed only in the Croatian group.

## 6 Conclusion

This study provides a cross‐national overview of the lived experience of parents of children with CAs in Europe. Our findings highlight a lack of support from HCPs, particularly in relation to psychological support at the time of diagnosis. Parents' experiences of support from HCPs differed across countries, with parents from Germany reporting the greatest satisfaction with support, and parents in Poland, Croatia and the United Kingdom generally reporting poor support. In contrast, support from friends, family and peers was consistently rated more positively across countries. This suggests that differences in parental experiences are more likely due to variations in medical systems as opposed to parental attitudes towards support. Our findings underscore the need to integrate psychosocial care into routine clinical pathways, improve training for HCPs in empathetic communication, and address the broader support needs of families, including within schools. Future research should focus on identifying effective strategies to improve the provision of support to ensure that all families, regardless of country or diagnosis, receive the care and support they need.

## Author Contributions

A.L.B. conceptualised the study. A.L.B., E.M., J.K.M. and J.R. contributed to the study design and survey development. E.M. and J.K.M. analysed the data. A.J.D., I.B., C.C.C., E.D.H., E.G., L.G., A.J.S., L.R.L., C.M.D., E.M., C.N.P., A.J.N., A.N., L.O., A.P. and A.R. oversaw the translation of the survey, managed local ethical approval of the protocol and recruited participants. E.M. drafted the manuscript. All authors contributed to, critically revised and approved the final manuscript.

## Ethics Statement

This research was performed in accordance with the Declaration of Helsinki and ethics approval for the overall study was granted by the St George's (University of London) Research Ethics Committee on 18^th^ December 2020 (reference number: 2020.0311). In Poland, ethics approval was granted on 10^**th**^ December 2020 by the Bioethics Committee at the Poznań University of Medical Sciences (reference number: 882/20). In Croatia, ethics approval was granted on 10^th^ December 2020 by the Ethics Committee of the Children's Hospital Zagreb (Protocol No: 02‐23/43‐1‐20 Zagreb). In Spain, ethics approval was granted on 21^st^ December 2020 by the Clinical Investigation Ethics Committee of the ‘Dirección General de Salud Pública y Centro Superior de Investigación en Salud Pública’ (reference number: 20201221/05). In Belgium, ethics approval was granted on 1^st^ March 2021 by the Ethics Committee of the University Hospital of Antwerp (reference: 21/06/084). In Portugal, ethics approval was granted on 16^th^ March by the Ethics Committee of the National Institute of Health Doutor Ricardo Jorge (CES‐INSA). In Germany, ethics approval was granted on 15^th^ April 2021 by the Medical Faculty of the Otto‐von‐Guericke‐University Magdeburg Research Ethics Committee (reference number: 44/21). In Italy, ethics approval was granted on 14^th^ June 2021 by the Research Ethics and Integrity Committee of the National Research Council Institute of Clinical Physiology in Pisa (CNR‐IFC) (protocol number 0065527/2019). No further local ethics approvals were required in Denmark (Lillebaelt Hospital—University Hospital of Southern Denmark) or the Netherlands (University Medical Center Groningen). All participants who took part in the survey provided informed consent.

## Conflicts of Interest

The authors declare no conflicts of interest.

## Supporting information


**Table S1:** Proportion* of participants reporting that they were ‘very satisfied’ with the support they received from each source, by congenital anomaly group.
**Table S2:** STROBE Statement—Checklist of items that should be included in reports of cross‐sectional studies.
**Figure S1:** Proportion* of participants reporting that they would have liked to have received psychological support at diagnosis with 95% confidence intervals, by country.

## Data Availability

The datasets analysed during the current study are available from the corresponding author on reasonable request.
