# Supplementary material for: Support Needs of Parents of Children With Congenital Anomalies Across Europe: A EUROlinkCAT Survey
Source: Child Care Health Dev. 2025 Sep 5;51(5):e70160. doi: 10.1111/cch.70160 (PMC12411889; doi:10.1111/cch.70160)
Supplement: Supplementary file 1 — Table S1: Proportion* of participants reporting that they were ‘very satisfied’ with the support they received from each source, by congenital anomaly group. Table S2: STROBE Statement—Checklist of items that should be included in reports of cross‐sectional studies. Figure S1: Proportion* of participants reporting that they would have liked to have received psychological support at diagnosis with 95% confidence intervals, by country. [file CCH-51-e70160-s001.docx]

# Supplementary file

## Tables and Figures

Table S1 Proportion* of participants reporting that they were ‘very satisfied’ with the support they received from each source, by congenital anomaly group.

| **Country** | **GP**  **(N†=679)** | **Specialist doctor/nurse**  **(N†=743)** | **Partner**  **(N†=752)** | **Friends/family**  **(N†=778)** | **Parents of children with same condition**  **(N†=624)** | **Patient organisations**  **(N†=511)** | **Schools**  **(N†=315)** |
| --- | --- | --- | --- | --- | --- | --- | --- |
|  | **%** [95% CI] | **%** [95% CI] | **%** [95% CI] | **%** [95% CI] | **%** [95% CI] | **%** [95% CI] | **%** [95% CI] |
| Cleft lip | 30 [22-37] | 67 [60-74] | 79 [73-85] | 59 [52-67] | 80 [73-86] | 61 [51-71] | 31 [19-42] |
| Spina bifida | 39 [28-49] | 44 [34-54] | 70 [60-79] | 53 [42-63] | 55 [44-66] | 37 [26-49] | 45 [31-58] |
| CHD | 39 [33-45] | 56 [50-63] | 66 [60-72] | 53 [47-59] | 66 [59-72] | 52 [45-60] | 26 [17-35] |
| Down syndrome | 32 [25-38] | 36 [29-43] | 73 [67-80] | 61 [55-68] | 61 [53-68] | 48 [40-56] | 41 [30-51] |
| Down syndrome with CHD | 20 [8-32] | 40 [26-53] | 63 [50-78] | 62 [47-76] | 56 [41-71] | 37 [22-53] | 27 [5-50] |
| Heterogeneity between countries | p=0.086 | p<0.001 | p=0.044 | p= 0.369 | p=0.001 | p=0.026 | p= 0.119 |
| *Adjusted by parental age, education level and country. Unadjusted proportions are not included in this table.  †Total number of participants completing the item, excluding ‘not applicable’ responses. Missing data: GP (n=13), specialist doctor/nurse (n=19), partner (n=21), friends/family (n=16), parents of children with same condition (n=19), patient organisations (n=21), schools (n=47).  CI = confidence interval; GP = general practitioner; CHD – congenital heart defect | | | | | | | |

Table S2 STROBE Statement—Checklist of items that should be included in reports of cross-sectional studies

|  | Item No | Recommendation | Section in paper |
| --- | --- | --- | --- |
| **Title and abstract** | 1 | (*a*) Indicate the study’s design with a commonly used term in the title or the abstract | See title and abstract |
|  |  | (*b*) Provide in the abstract an informative and balanced summary of what was done and what was found | See abstract |
| Introduction | | | |
| Background/rationale | 2 | Explain the scientific background and rationale for the investigation being reported | Introduction, paragraph 2-3 |
| Objectives | 3 | State specific objectives, including any prespecified hypotheses | Introduction, paragraph 3 |
| Methods | | | |
| Study design | 4 | Present key elements of study design early in the paper | Methods, paragraph 1 |
| Setting | 5 | Describe the setting, locations, and relevant dates, including periods of recruitment, exposure, follow-up, and data collection | Methods, Data Collection sub-heading |
| Participants | 6 | (*a*) Give the eligibility criteria, and the sources and methods of selection of participants | Methods, Data Collection sub-heading |
| Variables | 7 | Clearly define all outcomes, exposures, predictors, potential confounders, and effect modifiers. Give diagnostic criteria, if applicable | Methods, paragraph 3-4 |
| Data sources/ measurement | 8* | For each variable of interest, give sources of data and details of methods of assessment (measurement). Describe comparability of assessment methods if there is more than one group | Methods, paragraph 3-4 |
| Bias | 9 | Describe any efforts to address potential sources of bias | n/a |
| Study size | 10 | Explain how the study size was arrived at | Methods, Statistical analysis sub-heading |
| Quantitative variables | 11 | Explain how quantitative variables were handled in the analyses. If applicable, describe which groupings were chosen and why | Methods, Statistical analysis sub-heading |
| Statistical methods | 12 | (*a*) Describe all statistical methods, including those used to control for confounding | Methods, Statistical analysis sub-heading |
|  |  | (*b*) Describe any methods used to examine subgroups and interactions | Methods, Statistical analysis sub-heading |
|  |  | (*c*) Explain how missing data were addressed | Methods, Statistical analysis sub-heading |
|  |  | (*d*) If applicable, describe analytical methods taking account of sampling strategy | n/a |
|  |  | (*e*) Describe any sensitivity analyses | n/a |
| Results | | | |
| Participants | 13* | (a) Report numbers of individuals at each stage of study—eg numbers potentially eligible, examined for eligibility, confirmed eligible, included in the study, completing follow-up, and analysed | Results, paragraph 1 |
|  |  | (b) Give reasons for non-participation at each stage | n/a |
|  |  | (c) Consider use of a flow diagram | n/a |
| Descriptive data | 14* | (a) Give characteristics of study participants (eg demographic, clinical, social) and information on exposures and potential confounders | Results, paragraph 1-3 |
|  |  | (b) Indicate number of participants with missing data for each variable of interest | Table S1 |
| Outcome data | 15* | Report numbers of outcome events or summary measures | n/a |
| Main results | 16 | (*a*) Give unadjusted estimates and, if applicable, confounder-adjusted estimates and their precision (eg, 95% confidence interval). Make clear which confounders were adjusted for and why they were included | Confounders: Methods, statistical analysis sub-heading Unadjusted estimates: Supplementary file (section E) |
|  |  | (*b*) Report category boundaries when continuous variables were categorized | n/a |
|  |  | (*c*) If relevant, consider translating estimates of relative risk into absolute risk for a meaningful time period | n/a |
| Other analyses | 17 | Report other analyses done—eg analyses of subgroups and interactions, and sensitivity analyses | n/a |
| Discussion | | | |
| Key results | 18 | Summarise key results with reference to study objectives | Discussion, paragraph 1 |
| Limitations | 19 | Discuss limitations of the study, taking into account sources of potential bias or imprecision. Discuss both direction and magnitude of any potential bias | Discussion, limitations sub-heading |
| Interpretation | 20 | Give a cautious overall interpretation of results considering objectives, limitations, multiplicity of analyses, results from similar studies, and other relevant evidence | Discussion, implications sub-heading |
| Generalisability | 21 | Discuss the generalisability (external validity) of the study results | Discussion, implications sub-heading |
| Other information | | | |
| Funding | 22 | Give the source of funding and the role of the funders for the present study and, if applicable, for the original study on which the present article is based | Funding sub-heading |

*Give information separately for exposed and unexposed groups. **Note:** An Explanation and Elaboration article discusses each checklist item and gives methodological background and published examples of transparent reporting. The STROBE checklist is best used in conjunction with this article (freely available on the Web sites of PLoS Medicine at http://www.plosmedicine.org/, Annals of Internal Medicine at http://www.annals.org/, and Epidemiology at http://www.epidem.com/). Information on the STROBE Initiative is available at www.strobe-statement.org.

Figure S1 Proportion* of participants reporting that they would have liked to have received psychological support at diagnosis with 95% confidence intervals, by country.


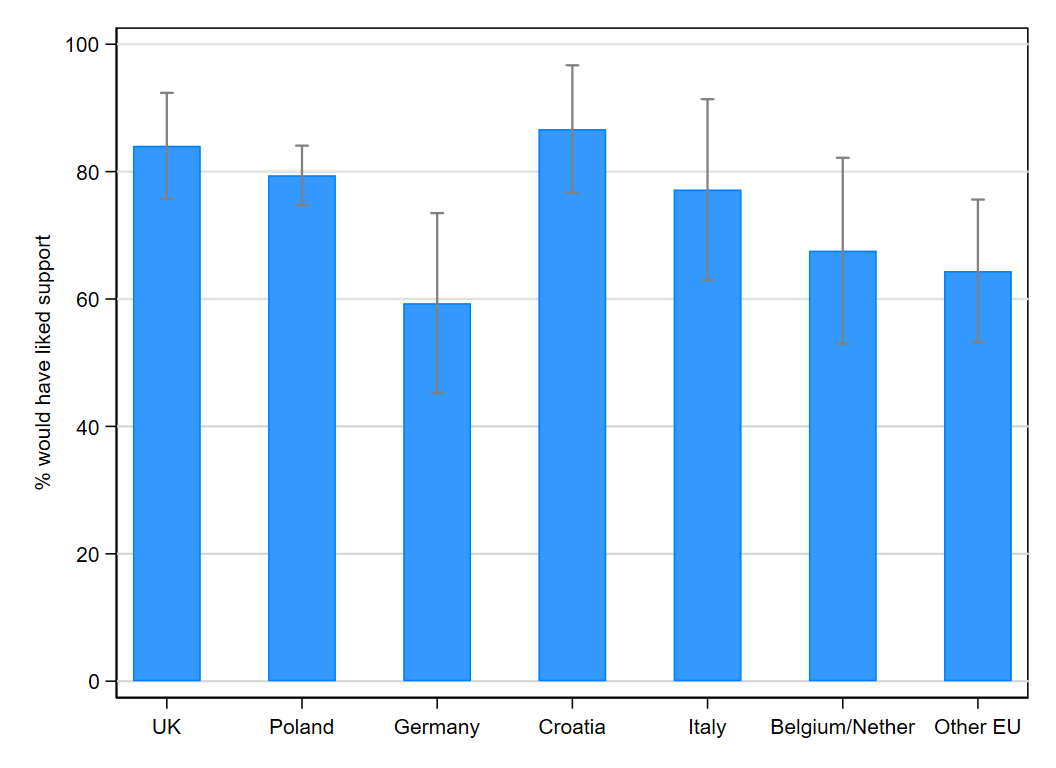


*Adjusted by congenital anomaly type, parental age, and education level.

## (B) Survey Items

### (1) Parent Demographics

1. Which country do you live in? [drop-down list]

2. What type of area do you live in?

1. City (population over 500,000)
2. Large town (population between 100,000 and 500,000)
3. Medium town (population between 20,000 and 100,000)
4. Small town (population less than 20,000)
5. Suburban village
6. Village
7. Rural/isolated area (e.g. a farm)

3. What is your age?

1. Less than 20 years
2. 20-25 years
3. 26-30 years
4. 31-35 years
5. 36-40 years
6. 41-45 years
7. 46-50 years
8. More than 50 years

4. What is the highest level of education you have completed?

1. Primary school
2. Secondary school up to 16 years
3. Secondary or further education after 16 years
4. University
5. Post-graduate / Doctoral studies

5. What is your employment status?

1. Employed (full-time), including self-employed
2. Employed (part-time), including self-employed
3. Full-time homemaker/carer
4. Long-term sick/disabled
5. Retired
6. Student
7. Unemployed
8. On furlough

6. How long have you lived in your country of residence?

1. Up to 1 year
2. Between 1-5 years
3. Between 6-10 years
4. More than 10 years
5. From birth
6. Prefer not to say

7. What is your relationship to the child this survey is about?

1. Mother (biological)
2. Mother (adoptive)
3. Father (biological)
4. Father (adoptive)
5. Legal guardian related to the child
6. Legal guardian unrelated to the child / foster parent
7. Another family member

### (2) Child Demographics and Medical Information

1. What age is your child?

1. Less than 1 year
2. 1-3 years
3. 4-6 years
4. 7-10 years

2. What is your child's gender?

1. Male
2. Female
3. Other
4. Prefer not to say

3. Which of the following conditions has your child been diagnosed with? (If your child has more than one of these conditions, please select all that apply)

1. Cleft lip (with or without cleft palate)
2. Spina bifida
3. Congenital heart defect that required surgical intervention
4. Down’s syndrome

4. Was your child’s [*condition*] detected prenatally (during pregnancy)?

1. Yes [survey moves to question 5]
2. No [survey skips to question 6]
3. I don’t know [survey skips to question 6]

5. In which week of pregnancy was your child’s [*condition*] detected?

1. Before 13 weeks
2. Between 14 and 21 weeks
3. At 22 weeks or later
4. I’m not sure

6. Does your child have any other congenital anomalies (conditions present from birth)?

1. Yes

Please select all that apply:

- Brain anomalies
- Hydrocephalus
- Eye anomalies
- Anomalies of face, ear and neck
- Lung anomalies
- Abdominal anomalies
- Renal anomalies
- Genital anomalies
- Skeletal anomalies
- Limb anomalies
- Chromosomal or genetic abnormality (other than Down’s syndrome)
- Other anomaly

1. No

7. Does your child have any other health conditions?

1. Yes

Please select all that apply:

- Autism or attention disorder
- Learning disability
- Epilepsy
- Cerebral Palsy
- Asthma
- Allergy or food intolerance
- Eczema or other skin disease
- Recurrent infections
- Hearing loss
- Vision problems
- Celiac disease
- Diabetes
- Endocrine disorder
- Immune disorder
- Blood disorder
- Cancer
- Other

1. No

### (3) Support at diagnosis

In the first month after your child’s diagnosis:

1. Did you receive support from the healthcare professionals treating your child?

1. **Not at all**
2. **A little**
3. **Quite a bit**
4. **Very much**
5. Not applicable (e.g. we are adoptive parents/foster family) [survey skips to question 4]

2. Did you receive support from friends and family?

1. **Not at all**
2. **A little**
3. **Quite a bit**
4. **Very much**

3. Did you receive any professional psychological support (e.g. from a psychologist or counsellor)?

1. No, I did not need professional support
2. No, but I would have liked professional support
3. Yes, I received free psychological support
4. Yes, I paid for private psychological support

### (4) Support after diagnosis (before the COVID-19 pandemic)

Thinking back to your experience before COVID-19:

4. To what extent did you feel satisfied with the support you received from the following people/organisations? (If you did not access or seek support from a listed source please select N/A)

|  | **Not at all satisfied** | **Slightly satisfied** | **Moderately satisfied** | **Very satisfied** | **N/A** |
| --- | --- | --- | --- | --- | --- |
| General practitioner |  |  |  |  |  |
| Specialist doctor or specialist nurse |  |  |  |  |  |
| School |  |  |  |  |  |
| Partner (or person I am closest to) |  |  |  |  |  |
| Friends and family |  |  |  |  |  |
| Parents of children with the same health condition |  |  |  |  |  |
| Patient/parent organisation |  |  |  |  |  |

5. Overall, would you have liked more support?

1. **Not at all**
2. **A little**
3. **Quite a bit**
4. **Very much**

## (C) Organisations supporting recruitment

The following organisations supported the recruitment of participants across Europe:

Spina Foundation (Poland), Borys the Hero Foundation (Poland), Fundacja TAK dla Samodzielności (Poland), Uniwersytet Medyczny im. Piastów Śląskich we Wrocławiu (Poland), Collegium Medicum Uniwersytetu Mikołaja Kopernika (Poland), The Cleft Lip and Palate Association (UK), The Children's Heart Federation (UK), Children's Heartbeat Trust (UK), Down's Syndrome Association (UK), Down Syndrome International (UK), A.S.B.I. Associazione Spina Bifida Italia (Italy), Fondazione Toscana Gabriele Monasterio (Italy), Associazione “Un cuore, un mondo” (Italy), Associazione “Trisomia 21 Onlus” (Italy), Azienda Ospedaliero Universitaria Pisana (Italy), Arbeitsgemeinschaft Spina Bifida und Hydrocephalus (Germany), University Hospital Magdeburg (Germany), Hjerteforeningens børneklub (Denmark), Rygmarvsbrokforeningen (Denmark), Downs syndrom Danmark (Denmark), Landsforeningen Læbe- Ganespalte (Denmark), Pais21 (Portugal), Associação Spina Bifida e Hidrocefalia de Portugal (Portugal), Associação Coração Feliz (Portugal), Associação Portuguesa dos Amigos das Crianças Portadoras de Fendas Lábio-Palatinas (Portugal), The Foundation for the Promotion of Health and Biomedical Research of Valencia Region (FISABIO, Spain), Vereniging voor Aangeboren Gelaatsafwijkingen, VAGA (Belgium), Centrum voor Ontwikkelingsstoornissen, COS (Belgium), Spina Bifida Hydrocephalus Belgium, International Federation for Spina Bifida and Hydrocephalus (Belgium), Universitair Ziekenhuis Antwerpen (Belgium), Hrvatski savez za rijetke bolesti (Croatia), Veliko srce malom srcu (Croatia), Hrvatska zajednica za Down sindrom (Croatia), Udruga roditelja djece s rascjepom usne i/ili nepca OSMIJEH (Croatia), Udruga Aurora- Udruga roditelja i djece sa spinom bifidom (Croatia), Patientenvereniging Aangeboren Hartaandoeningen (Croatia), De 'Stichting Downsyndroom' (Croatia).

## (D) Unadjusted frequencies of responses to survey items (full sample)

### Support at diagnosis

**In the first month after your child’s diagnosis…**

Table 3 … did you receive support from the healthcare professionals treating your child?

| Response options | Frequency | Percent |
| --- | --- | --- |
| Not at all | 154 | 15.65 |
| A little | 312 | 31.71 |
| Quite a bit | 239 | 24.29 |
| Very much | 262 | 26.63 |
| N/A | 17 | 1.73 |
| Total | 984 | 100.00 |

Table 4 … did you receive support from friends and family?

| Response options | | Frequency | Percent |
| --- | --- | --- | --- |
| Not at all | | 43 | 4.45 |
| A little | 197 | | 20.39 |
| Quite a bit | 257 | | 26.60 |
| Very much | 469 | | 48.55 |
| Total | 966 | | 100.00 |

Table 5 … did you receive any professional psychological support?

| Response options | Frequency | Percent |
| --- | --- | --- |
| Did not need support | 344 | 35.87 |
| Would have liked support | 468 | 48.80 |
| Received free support | 111 | 11.57 |
| Paid for private support | 36 | 3.75 |
| Total | 959 | 100.00 |

### Support after diagnosis

To what extent did you feel satisfied with the support you received from the following people/organisations?

Table 6 General practitioner

| Response options | Frequency | Percent |
| --- | --- | --- |
| Not at all satisfied | 126 | 15.04 |
| Slightly satisfied | 123 | 14.68 |
| Moderately satisfied | 204 | 24.34 |
| Very satisfied | 232 | 27.68 |
| N/A | 153 | 18.26 |
| Total | 838 | 100.00 |

Table 7 Specialist doctor or nurse

| Response options | Frequency | Percent |
| --- | --- | --- |
| Not at all satisfied | 59 | 7.09 |
| Slightly satisfied | 100 | 12.02 |
| Moderately satisfied | 211 | 25.36 |
| Very satisfied | 380 | 45.67 |
| N/A | 82 | 9.86 |
| Total | 832 | 100.00 |

Table 8 Partner (or person you are closest to)

| Response options | Frequency | Percent |
| --- | --- | --- |
| Not at all satisfied | 33 | 3.98 |
| Slightly satisfied | 46 | 5.54 |
| Moderately satisfied | 139 | 16.75 |
| Very satisfied | 540 | 65.06 |
| N/A | 72 | 8.67 |
| Total | 830 | 100.00 |

Table 9 Friends and family

| Response options | Frequency | Percent |
| --- | --- | --- |
| Not at all satisfied | 31 | 3.71 |
| Slightly satisfied | 103 | 12.34 |
| Moderately satisfied | 203 | 24.31 |
| Very satisfied | 447 | 53.53 |
| N/A | 51 | 6.11 |
| Total | 835 | 100.00 |

Table 10 Parents of other children

| Response options | Frequency | Percent |
| --- | --- | --- |
| Not at all satisfied | 28 | 3.37 |
| Slightly satisfied | 55 | 6.61 |
| Moderately satisfied | 134 | 16.11 |
| Very satisfied | 412 | 49.52 |
| N/A | 203 | 24.40 |
| Total | 832 | 100.00 |

Table 11 Parent/patient organisations

| Response options | Frequency | Percent |
| --- | --- | --- |
| Not at all satisfied | 42 | 5.06 |
| Slightly satisfied | 52 | 6.27 |
| Moderately satisfied | 166 | 20.00 |
| Very satisfied | 255 | 30.72 |
| N/A | 315 | 37.95 |
| Total | 830 | 100.00 |

Table 12 School

| Response options | Frequency | Percent |
| --- | --- | --- |
| Not at all satisfied | 53 | 6.59 |
| Slightly satisfied | 55 | 6.84 |
| Moderately satisfied | 101 | 12.56 |
| Very satisfied | 109 | 13.56 |
| N/A | 486 | 60.45 |
| Total | 804 | 100.00 |

Table 13 Overall, would you have liked more support?

| Response options | Frequency | Percent |
| --- | --- | --- |
| Not at all | 83 | 9.92 |
| A little | 231 | 27.60 |
| Quite a bit | 225 | 26.88 |
| Very much | 298 | 35.60 |
| Total | 837 | 100.00 |
